# Clinical Perspectives on the Histomolecular Features of the Pancreatic Precursor Lesions: A Narrative Review

**DOI:** 10.34172/mejdd.2024.387

**Published:** 2024-07-31

**Authors:** Sayedeh-Zahra Kazemi-Harikandei, Amirali Karimi, Seyed Mohammad Tavangar

**Affiliations:** ^1^School of Medicine, Tehran University of Medical Sciences, Tehran, Iran; ^2^Department of Pathology, Shariati Hospital, Tehran University of Medical Sciences, Tehran, Iran; ^3^Chronic Diseases Research Center, Endocrinology and Metabolism Population Sciences Institute, Tehran University of Medical Sciences, Tehran, Iran

**Keywords:** Pancreatic cystic lesions (PCL), Pancreatic ductal adenocarcinoma, Intraductal papillary mucinous neoplasms (IPMN), Pancreatic intraepithelial neoplasms (PanIN), Mucinous cystic neoplasms (MCN), Early detection of cancer, Targeted therapy

## Abstract

Pancreatic cancer (PC) is a lethal cancer with poor prognoses. Identifying and characterizing pancreatic cystic lesions (PCLs) in the early detection and follow-up plans is thought to help detect pancreatic malignancy. Besides, the molecular features of PCLs are thought to unravel potentials for targeted therapies. We present a narrative review of the existing literature on the role of PCLs in the early detection, risk stratification, and medical management of PC. High-grade intraductal papillary mucinous neoplasms (IPMN) and pancreatic intraepithelial neoplasia (PanIN) stage III are high-risk lesions for developing PC. These lesions often require thorough histomolecular characterization using endoscopic ultrasound (EUS), before a surgical decision is made. EUS is also useful in the risk assessment of PCLs with tentative plans–for instance, in branch-duct IPMNs (BD-IPMN)- where the final decision might change. Besides the operative decisions, recent improvements in the application of targeted therapies are expected to improve survival measures. Knowledge of molecular features has helped develop targeted therapies. In summary, the histomolecular characterization of PCLs is helpful in optimizing management plans in PC. Further improvements are still needed for the broad application of this knowledge in the clinical setting.

## Introduction

 Pancreatic cancer (PC) is the seventh leading cause of cancer mortality worldwide.^1^ Achieving an earlier diagnosis and implementing more effective oncological treatments are two significant problems that need to be addressed in tackling the current low survival rates.^2^ Targeting early neoplastic lesions seems beneficial due to the long window period before developing non-resectable lesions.^3^

 Intraductal papillary mucinous neoplasms (IPMN), mucinous cystic neoplasm (MCN), and pancreatic intraepithelial neoplasia (PanIN) are the main precursor lesions to the pancreatic ductal adenocarcinoma (PDAC)^4^–called PC in this paper. Pancreatic cystic lesions (PCLs) possess specific histological and molecular characteristics that have both diagnostic and prognostic significance. The diagnostic markers include histological features for resemblance to other gastrointestinal cancers –for instance, in IPMNs- and the expression of surface markers –glycoprotein mucin 1 to 6 (MUC1-6) and estrogen and progesterone receptors (ER and PR).^4^ The genetic landscape of each lesion is the most direct indicator for determining the risks of malignant conversion for PCLs. Using specific genetic aberrations, including KRAS, TP53, p16/CDKN2A, and SMAD4, enables the differentiation of high-risk neoplastic lesions.^5,6^ Moreover, the designation of the precursor lesions based on their histomolecular findings has helped determine the preoperative decisions and follow-up plans. Thus, using detailed histomolecular profiles from the precursor neoplastic lesions is expected to improve personalized therapeutic and surveillance decisions in PC.^7^

 Currently, several points of debate exist in the management of PCLs. Endoscopic ultrasound (EUS) is a study of choice in cases where the initial imaging studies are not definitive.^8^ EUS enables the direct visualization of the lesion connections to the ductal systems. Additionally, providing a biopsy or fine-needle aspiration (FNA) helps determine the extensive histological and molecular characteristics preoperatively.^9^ Operative management is often a good choice in cases of PCLs with considerable malignancy risks.^10^ Nonetheless, the decision is often problematic in lesions with relatively close risks and benefits. According to the recent version of the European evidence-based guidelines on PCLs, main-duct IPMN (MD-IPMN) and mixed-type IPMN should be managed surgically in most cases (see below). On the other hand, branch-duct IPMNs (BD-IPMN) are mainly managed conservatively, with frequent imaging studies for follow-up.^8^ As mentioned, the obtained data regarding the histomolecular characteristics of the lesion is also helpful in determining follow-up plans. Notably, several non-invasive methods are under investigation for early detection and risk assessment in pancreatic neoplasms. In this regard, liquid biopsy is appealing as it provides access to genetic findings while minimally invasive.^2^

 Currently, there is no effective treatment in patients with unresectable pancreatic lesions.^10^ This is while molecular targeted therapies and immunotherapies are being used in the setting of several cancer types.^11^ Additionally, several benefits have been observed in the earlier phase studies of PC. Most recently, Pant and colleagues demonstrated significant tumor biomarker response to KRAS-specific amphiphile vaccine for patients with pancreatic malignancy.^12^ Knowledge of the molecular alterations in PCLs and their application in clinical settings have been previously demonstrated to improve clinical outcomes.^13^ However, there are debates regarding when to use the molecular characterization of these samples.^14^ A closer look at the present guidelines is helpful in improving management plans for PC.

 In summary, histomolecular findings of PCLs entail invaluable data regarding early diagnosis, determining prognosis, and directing treatment plans for pancreatic malignancy. In this review, we tried to demonstrate the most relevant features of PCLs and their applications in the clinical setting of PC. We aimed to depict the existing pitfalls in clinical decision-making for these pancreatic neoplasms (Table 1).

**Table 1:** Classical and current clinical applications of the histological and molecular features of pancreatic cystic lesions based on the type of application

**Clinical utility**	**Classical application**	**Novel application**
Diagnosis	Tissue resection is the gold standard method in characterizing the lesions. Based on the European guidelines^8^: - The absolute indications for resection are IPMN with positive cytology for malignancy/high-grade dysplasia, solid mass, jaundice (tumor-related), enhancing mural nodules ( ≥ 5 mm), MPD dilation ( ≥ 10 mm) - The relative indications for resection are IPMN with growth rate ≥ 5 mm/year, increased serum CA19-9 level ( > 37 U/mL in the absence of jaundice), MPD diameter of 5-9.9 mm, cyst diameter ≥ 40 mm, symptoms (new-onset of diabetes mellitus or acute pancreatitis), and contrast-enhancing mural nodules < 5 mm. EUS and FNA are indicated and used in all patients with PCLs and the above-mentioned relative indications, also known as “worrisome features”.	Despite its broad application, singular cytologic findings from EUS and FNA have not been sensitive in detecting PCLs.^15^ Most recently, EUS-guided through-the-needle biopsy for pancreatic cysts has been shown to be sensitive and highly specific for identifying malignant pancreatic lesions.^16^
Risk assessment	KRAS and GNAS mutations are associated with a greater risk of malignancy, differentiating high-risk IPMN from MCN.^17^ MUC2 and intestinal markers can be used to differentiate PanIN from intestinal IPMN and change management plans.^18^	Greater miR-21 and miR-155 levels have been observed in invasive compared with non-invasive IPMNs.^19^ cfDNA has been shown effective in identifying PC, both qualitative and quantitatively – by KRAS, GNAS, and TP53 levels.^20^
Intra-operative decision	The presence of high-grade IPMN on the resection margin mandates repeat resection.^21^ PanIN III on the intraoperative frozen section needs further resection.^4^	Intraoperative molecular characterization of the resection sites -for instance, for CEA- has shown effectiveness in detecting PC tissue.^22^
Medical management	Gemcitabine and similar chemotherapeutic options in high-risk IPMN and MCN have been used in PCL clinics.^23^	Pant et al. demonstrated the usefulness of the KRAS-specific amphiphile vaccine, which was shown as a tumor biomarker response.^12^
Follow-up	AGA supports the discontinuation of follow-up in cases with low-risk IPMNs.^24^ Large MCN lesions are often managed surgically to spare individuals from long-term follow-up. Nonetheless, close follow-up is required in cases with high-grade MCNs.^25^	Liquid biopsy markers are thought to be useful minimally invasive follow-up tools for PCLs. cfDNA has been previously shown as an effective prognostic measure, both qualitative and quantitatively - by KRAS mutations.^26^
Early detection	Previously, the focus has been on persons with hereditary pancreatitis, cystic fibrosis, or familial history of malignancies – i.e. PC, colorectal cancer- and specific mutations –BRCA1 and 2. AGA has pinpointed high-grade PCLs -namely PanIN III and high-grade IPMN- as the main targets for follow-up screening studies.^27^	The role of circulating nucleic acid markers and CTCs has been emphasized in the screening of precursor lesions for the development of PC.^28,29^

AGA: American Gastrointestinal Association, CEA: carcinoembryonic antigen, cfDNA: cell-free DNA, CTC: circulating tumor cell, EUS: endoscopic ultrasound, FNA: fine-needle aspiration, IPMN: intraductal papillary mucinous neoplasm, MCN: mucinous cystic neoplasm, MPD: main pancreatic duct, PanIN: pancreatic intraductal neoplasm, PC: pancreatic cancer, PCL: pancreatic cystic lesion, SCN: serous cystic neoplasm.

## A Histomolecular Characteristics

 IPMNs are among the most common precursor lesions to pancreatic malignancy.^30^ There are several classification methods for IPMNs, considering their histological and molecular features. Classically, IPMNs have been subcategorized as gastric, intestinal, pancreatobiliary, and oncocytic microscopic subtypes. This classification is mainly based on the histological features and resemblance to other cancer subtypes.^31^ Specifically, gastric IPMNs are lesions with the most benign behaviors –associated with low-grade dysplasia. These lesions are mostly attached to the pancreatic branch duct (BD). Together with the pancreatobiliary IPMNs, these lesions are precursors to the tubular subtype of pancreatic ductal carcinoma.^24^ Both gastric- and pancreatobiliary-IPMN express MUC5AC; however, only the latter group demonstrates MUC1. Intestinal IPMN is another subgroup with a more distinct course. These lesions demonstrate moderate- to high-grade dysplasia and are mainly attached to the main pancreatic duct (MPD).^32^ The intestinal subtype presents MUC5AC, and intestinal-differentiation markers, MUC2 and CD-X.^5,33^ These latter markers, along with GNAS mutation, are helpful for their differentiation from other PCLs (see below). Oncocytic IPMNs have the highest risks of malignancies among all IPMNs.^4^ Nonetheless, due to their different pathologic pathway and rare presentation, they are not discussed in this paper.

 The risk of malignancy is the lowest in gastric-type and rises from the intestinal to pancreatobiliary, respectively.^24^ *KRAS *and* GNAS* mutations are the most common molecular findings in IPMNs, mostly occurring in gastric- and intestinal subtypes, respectively. KRAS, p16/CKN2A, TP53, and SMAD4 are the associated markers for invasiveness.^34^ Another type of categorization for IPMNs is based on their connectedness with the MPD and BDs. Accordingly, MD-IPMN and mixed-type IPMN are connected to the main duct, while BD-IPMN is only connected to the branch duct. MD-IPMN harbors the most significant risk of malignancy –about 30%. On the other hand, BD-IPMNs have variable risks of malignancy, and their management depends on the presence of “worrisome features,” which are mentioned later.^21^ The most recent version of the classification in IPMNs is from the Baltimore Consensus Meeting.^35^ In their report, it has been recommended that IPMNs should only be presented as low- or high-grade lesions, with the possible presence of concurrent malignant tissue.

 MCN mainly occurs in middle-aged women. They are often found as large cystic lesions ( > 3 cm), not communicating with the MPD. Due to this anatomy, they must be differentiated from serous cystic neoplasms (SCNs) and BD-IPMNs.^31^ Imaging is often valuable in this regard. Currently, the identification of invasive MCN tissue is challenging. Histologically, MCNs express MUC1, MUC2, and MUC5AC, as well as the hormone receptors (ER, PR). The basic molecular alterations found in MCN are KRAS and ring finger protein 43 (RNF43). Furthermore, high-grade lesions demonstrate loss of CDKN2A, TP53, and SMAD4.^36^ Due to the paucity of specific genetic markers for MCN, the preoperative diagnosis is based solely on excluding other differential diagnoses.^37^ The 5-year survival rates vary from nearly 100% in individuals with low-grade to about 60% in those with high-grade MCNs. With the lack of differentiating markers for malignancy, this trend has favored overtreatment in the management of MCN (see below).^9,38^ This is partly due to the extensive sampling requirements and unreasonably high follow-up costs. Accordingly, the management of choice is surgical resection in younger individuals with cysts greater than 4 cm.^8,9^ Nonetheless, there have been debates related to the threshold for needs of surgery - surveillance recommended for cysts less than 3 cm without high-risk features by AGA - or a need for surgery for all MCN lesions - as recommended by the 2017 International Consensus guideline.^39^

**Figure 1:**
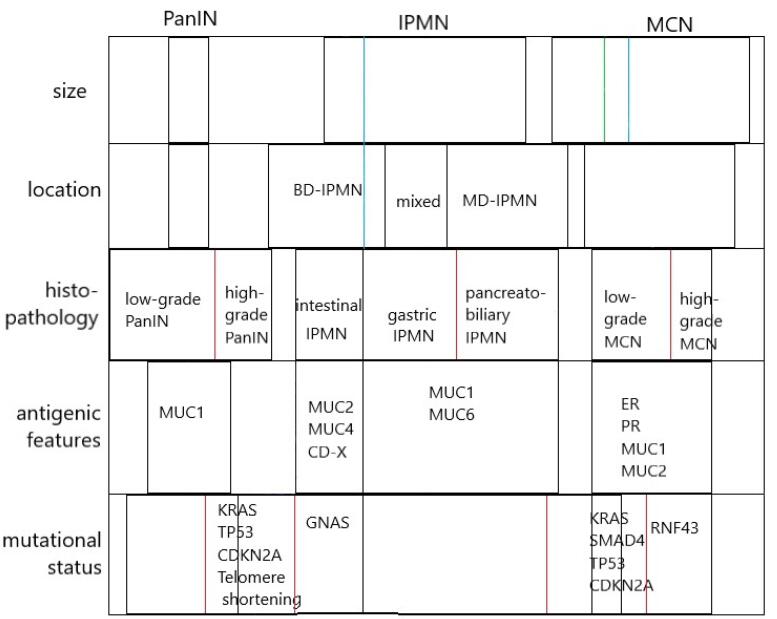
An overview of the presented applications of the histomolecular characteristics in the clinical setting of pancreatic cystic lesions (PCLs). As indicated, the three most common groups of PCLs are represented –namely intraductal papillary mucinous neoplasms (IPMN), mucinous cystic neoplasms (MCN), and pancreatic intraepithelial neoplasms (PanIN). The rows demonstrate the category of the pathological finding. The columns represent the three lesion types in a spectrum within each pathologic finding. The lesions are separated in each category by the histological and molecular findings, with red lines indicating risks of malignancy, blue lines related to the indication of surgical management, and green lines for follow-up discontinuation. BD: branch-duct, MD: main-duct, MUC: glycoprotein mucin receptor, ER: estrogen receptor, PR: progesterone receptor

 PanINs are microscopic lesions commonly found on PC surgical resections. Their characteristic molecular and histological features delineate the progression markers of ductal adenocarcinoma. Molecular features include KRAS mutation followed by CDKN2A/p16, SMAD4, and TP53 as late events. PanINs are not identifiable in routine imaging studies and are identified retrospectively in most cases.^40^ Traditionally, PanINs have been categorized into a three-tier system with PanIN stage III, also called carcinoma in situ lesions.^35^ More recently, PanIN I and II were debated to be clinically significant; after that, PanIN has been recommended to be mentioned as low-grade and high-grade lesions. In this classification, the former PanIN III is considered high-grade PanIN, and its importance is due to the presented risks of malignancy.^41^ EUS-based FNA studies have enabled the preoperative identification of these lesions.^8^ These lesions express MUC1 and are associated with the markers of malignancy –including KRAS, telomere shortening, TP53, and CDKN2A.^4^ Currently, the only markers for differentiating BD-IPMNs from PanIN (and MCNs) are the intestinal markers, MUC2 and CD-X. Nonetheless, practical diagnostic guidelines for PanINs are still lacking, as these lesions are often found adjunct to more important malignant tissues (Figure 1).

## Clinical Remarks from the Histomolecular Findings

###  Initial Work-up

 The initial investigation for an individual with a suspected pancreatic lesion – based on clinical or laboratory findings – includes conventional imaging. For lesions with raised suspicions of malignancy – named as “worrisome features” as mentioned in the European guideline 2018- EUS-based studies are indicated.^8^ Pancreatic juice cytology identified by endoscopic retrograde cholangiopancreatography helps separate the management of lesions with “worrisome features”. In a retrospective study by Ohtsuka and others, the obtained cytological remarks could change the management plans in several cases with accuracy greater than conventional imaging.^42^ Specifically, pancreatic juice cytology demonstrated 94% accuracy for identifying “worrisome features” and changed the management plan in 5 out of 29 individuals who were not identified as high-risk based on the imaging. Due to the expensiveness and invasiveness, molecular characterization of cyst fluid samples remains limited to places where additive.^9,30^ Other modalities with less invasive pathological sampling include EUS-cytological brushing, which is more accurate and safer than FNA. As mentioned by Al-Haddad and colleagues, this method demonstrates acceptable rates of adverse events and is significantly more potent in identifying cystic features than the conventional FNA.^43^ However, these studies lack sufficient evidence and are currently limited to research fields.^8^

###  Pre-op Decision

 One of the major debates in the premalignant management of PCLs is determining risk-benefits for surgical plans. Tissue resection is the current gold-standard method for depicting histomolecular profiles.^44^ However, as the proper grading is not attainable preoperatively, current risk stratification systems focus on the anatomical and imaging findings –per requirements.^45^ Accordingly, patients with IPMN and absolute indications for surgical resection include “positive cytology for malignancy/high-grade dysplasia, solid mass, jaundice (tumor-related), enhancing mural nodules ( ≥ 5 mm), MPD dilation ( ≥ 10 mm)”. Additionally, relative indications which should be considered based on the patient’s clinical condition are “growth rate ≥ 5 mm/year, increased serum CA19-9 level ( > 37 U/mL in the absence of jaundice), MPD diameter of 5-9.9 mm, cyst diameter ≥ 40 mm, symptoms (new-onset of diabetes mellitus or acute pancreatitis), and contrast-enhancing mural nodules < 5 mm”.^8^ As mentioned, the operative decision is seemingly straightforward in MD- and mixed-type IPMNs, while BD-IPMNs are more commonly considered for conservative management.

 Differentiating premalignant from non-mucinous lesions –i.e., SCNs- is crucial as they do not require aggressive management.^46^ The level of carcinoembryonic antigen (CEA) is the most accurate marker for distinguishing pancreatic cystic neoplasms. Soyer and colleagues demonstrated 89.5% and 73.6% accuracies for differentiating mucinous and non-mucinous lesions by CEA and CA72-4, respectively.^47^ Additionally, cytology obtained 6% and 20% additive predictive ability for the mentioned accuracies of the CEA and CA72-4. As mentioned by Thornton and co-workers, EUS-FNA has a pooled sensitivity of 54% and specificity of 93% for differentiating mucinous from non-mucinous lesions.^48^ Currently, there is a strong consensus over the analysis of cystic fluid for CEA and cytology with or without KRAS/GNAS mutation status in distinguishing the mucinous lesions.^8^ As mentioned by Singhi and others, KRAS/GNAS mutations improve the diagnostic ability for making such discrimination – an estimated 89% sensitivity and 100% specificity.^49^ Other markers included cystic fluid glucose level, with the best accuracy when combined with intracystic CEA levels.^50^

 Differentiating different subtypes of PCLs is another point of debate when making initial management plans. MCNs are among the differential diagnoses of BD-IPMN.^21^ These lesions are more commonly viewed as large solitary asymptomatic cystic lesions compared with IPMNs; however, conventional imaging lacks acceptable accuracy in identifying these two entities.^4^ Among the EUS-based imaging findings, one can exemplify the absence of dilation in the MPD in MCNs.^25^ *GNAS* mutation was previously shown to be helpful for the differentiation of IPMNs in this regard. A 100% specificity and 20-40% sensitivity were revealed.^17^ Nonetheless, the definitive differentiation between these two entities is only made by surgical exploration, which is confirmed by the presence of ovarian stroma for MCN diagnosis.^9^ Due to similarities in the morphologies, PanINs should also be distinguished from intestinal-IPMNs. As mentioned before, MUC2 and intestinal markers are helpful in this regard.^18^

###  Determining Risks of Malignancy and Invasiveness

 EUS-FNA is valuable for preoperative tissue characterizations and, thus, determining the risks of malignancy.^51^ Current guidelines support using EUS where specific worrisome features are observed in imaging studies. Using EUS has the additional benefit of demonstrating cyst features related to the pancreatic ductal system.^25,46^ In detail, specific features that might not be identifiable on conventional imaging, including changes in MPD width, presence of mural nodules, and wall enhancements, could be viewed. Thus, proper differentiation of high-risk lesions needing surgery, including MD-IPMN and MCN from BD-IPMN, is attainable.^9^

 As emphasized by the European Society for Medical Oncology clinical practice guideline, EUS with FNA sampling could have up to 95% diagnostic accuracy for PC detection.^10^ Among the IPMNs, gastric and pancreatobiliary subtypes are more commonly seen in BD-, whereas the intestinal subtype is associated with MD-IPMN. While the former group is associated with tubular adenocarcinoma and a poorer prognosis, the latter accompanies colloid PC with a more favorable prognosis.^52^ Tubular invasive IPMN histologically resembles conventional PDAC. Additionally, these lesions have comparable prognoses overall compared with singular PDAC lesions.^52^ However, invasive IPMN demonstrates better prognoses and less aggressive behaviors in such a comparison.^53^ Malignancy markers, including KRAS, TP53, and telomere shortening, have high sensitivity but low specificity for the detection of conventional PC.^54^ In a comparative study by Mas and colleagues, KRAS mutation was more commonly found in PC, followed by the tubular and colloid subtypes of invasive IPMN.^55^ GNAS mutation was found in the tubular subtype irrespective of the degree of dysplasia. TP53, SMAD4, and CDKN2A were associated with invasiveness and seen more commonly in the tubular subtype. In summary, these markers of malignancy could help differentiate the clinically distinct entities.

 While EUS-FNA cytology is widely known for differentiating malignant lesions in preoperative decisions, mounting studies have focused on specific molecular features for better differentiation.^8^ In the earliest studies, Tada and colleagues used EUS-FNA and tissue biopsy to specify a differentiation system for cancerous and non-cancerous lesions of the pancreata. Results demonstrated the beneficial role of molecular findings in the cytological results for identifying PC – 62%, 77%, and 81% sensitivity for cytology, KRAS mutation positivity, and combined results, respectively.^56^ In a meta-analysis by Nissim and colleagues, hTERT and Shh showed the strongest associations with higher grades of IPMNs – grade III.^57^ In a study by Suzuki and others, cytology results obtained by EUS-FNA helped distinguish malignancy in IPMN with sensitivity and specificity of 64.8% and 90.6%, respectively.^58^ Combined analysis methods seem effective in the current diagnostic methods for the preoperative decision of PCLs. In a retrospective study, Springer and co-workers demonstrated the effectiveness of combined molecular and clinical markers in identifying precursor lesions for PC – a 100% identification ability for detecting SCN.^59^ Moreover, 0% malignancy risk was observed for mucinous lesions predicted by the model as low- to moderate-invasive risk.

 One novel application of minimally invasive methods for PCLs has been EUS-guided through-the-needle biopsy. This method has been introduced as an alternative to pancreatic juice FNA cytology, with acceptable sensitivity and high specificity – 76.6% and 98.9%, respectively – for detecting malignant pancreatic lesions.^16^ Nonetheless, multiple adverse effects, including bleeding and pancreatitis, still limit its wider application.^60^

 A group of novel markers for the preoperative risk stratification of PCLs comprise nucleic acid tumor markers. Caponi and colleagues demarcated the role of several micro-RNAs as prognostic markers of IPMN. In their study, miR-21 and miR-155 levels were significantly higher in invasive IPMNs compared with non-invasive lesions. Additionally, higher levels of miR-21 were associated with poorer overall survival rates in individuals with an IPMN –hazard ratio of 2.47 and a confidence interval of 95%.^19^ Circulating tumor DNA (ctDNA) and cell-free DNA (cfDNA) belong to DNA-based tumor markers. These markers enable minimally invasive sampling to obtain blood-based and other liquid-based tumor characterization (see below).^61^ Previously, cfDNA was shown to be helpful for both qualitative and quantitative analysis of the genetic markers of PC.^26^ It was also the single most effective predictive marker for the prognosis of PC. Additionally, Takai and others depicted the usefulness of cfDNA in PC for identifying KRAS, GNAS, and TP53.^20^ As previously mentioned by our research group, several novel markers are now available for minimally invasive characterization of the tumors.^28^ However, issues related to the expensiveness and lack of validity exist that limit their wide usage.^26^

###  Intra-op Decision

 One challenge in the surgical management of IPMN is obtaining resections with negative margins. An intraoperative frozen section is obtained during the surgery for individuals with IPMN.^62^ In cases with positive IPMN on the resection site, it is often difficult to obtain an accurate grading intraoperatively based on the histological remarks. Accordingly, for high-grade or invasive lesions in the surgical margin, repeat resection should be performed.^21^ On the other hand, positive margins for non-invasive IPMNs are not associated with increased risks of recurrence in the site of origin, and further resection is not usually required.^25,63^ For PanINs, their presence in the intraoperative frozen section makes the management seemingly debatable. PanIN on the resection margin of PC was shown not to change the prognoses, whereas a positive margin for PC was associated with lower survival rates.^64^ Distler and colleagues recommended similar management for PanINs as for IPMNs on the resection sites; thus, further resection is only indicated in the case of PanIN III.^4^

###  Follow-up

 Follow-up decisions in IPMNs are based on the risks of developing malignancy. Hirono and colleagues indicated a 6% risk of recurrence in the remnant tissue in patients with IPMN over 5 years (median years of 39 months from surgery).^65^ Crippa and others investigated the effectiveness of conservative management in BD-IPMN with low-risk features. Results indicated low recurrence rates –about 2%- and low rates of malignancy-associated deaths in these individuals.^66^ In a similar study design, Pergolini and colleagues obtained findings indicating the need for long-term follow-up in individuals with low-risk BD-IPMN.^67^ This study implicated about 8% risk of malignancy in 10 years, suggesting that developing benign lesions might accompany more significant risks for developing malignancy and thus needs follow-up. Almost identical risks were obtained by Tanno and others for resected and conservatively managed BD-IPMNs – about 4% for developing concurrent and future PCs during 5 years of follow-up.^68^ Irrespective of the management plan, surveillance of BD-IPMN is recommended to be planned based on malignancy risks. Several guidelines recommend lifelong follow-up, while AGA suggests follow-up cessation after 5 years in the absence of changes in the cyst size. Importantly, the surveillance program for resected IPMNs should be adjusted based on dysplastic features.^2^

 While a low risk of recurrence rules out the need for invasive management, it still does not resolve the necessity for observing the individuals. Among the existing guidelines, only AGA supports discontinuation of follow-up in low-risk IPMNs not demonstrating any signs of progression over 5 years.^21,69^ Previous studies have demonstrated relatively low risks in low-grade MCN – almost 6% for cysts less than 3 cm- for concomitant PC in these cases.^70^ A close follow-up for about 2-5 years is required in individuals undergoing conservative management, with updating programs based on the clinical and imaging progression. Meanwhile, invasive MCN requires postoperative surveillance similar to that indicated for conventional PC.^25^ Notably, margin positivity is only clinically significant in certain cases and thus requires finding individuals with high-risk lesions.

 It has been hypothesized that concurrent pancreatic malignancy in individuals with the diagnosis of IPMN might have arisen from two origins: first, an invasive component from IPMN, and second, concurrent PDAC tissues with distinct origins. Due to the differences mentioned above in the clinical courses and survival rates, invasive IPMN and *de novo* PDAC should be adequately distinguished. Tamura and others have demonstrated that molecular markers, including KRAS/GNAS, help distinguish the different origins of these two lesions –an almost 89% discrimination performance.^71^

 Liquid biopsy markers are other predictors of malignancy that could be used in follow-up plans.^21^ In a study by Bunduc and colleagues, cfDNA – specifically with KRAS mutation- was shown to be an effective predictive marker of the survival rates in PDAC. Specifically, detectable ctDNA and KRAS mutation were associated with decreased progression-free survival with HR of 1.86 and 1.92, respectively. Additionally, overall survival was decreased in the presence of detectable ctDNA and KRAS mutation with 2.25 and 1.52 increased risks.^26^ Nonetheless, the role of such markers in prognosticating IPMN and other PCLs remains to be elucidated.

###  Early Detection

 As mentioned, early detection is one of the most significant challenges in improving survival rates in individuals with PC. The current focus in early detection of PC is mainly based on specific clinical conditions – hereditary pancreatitis, cystic fibrosis-, familial history of malignancies – PC, colorectal cancer- and specific mutations – BRCA1 and 2.^72^ According to a statement by the International Cancer of the Pancreas Screening in 2013, identifying PCLs was considered a successful screening program.^73^ More recently, in an expert review by Aslanian and others, precursor pancreatic lesions – especially high-grade IPMN and PanIN III- were stated as the main targets in the current screening attempts.^27^ Early detection of PC is mainly based on histological and molecular findings. EUS-guided tissue biopsy is considered a possible tool for the non-operative screening of high-risk individuals for PC.^74^ This tool has been complementary to conventional imaging in cases with smaller sizes and the presence of solid lesions.^75^

 Screening is not recommended for the detection of PCLs in asymptomatic, non-increased-risk individuals. Nonetheless, after the individuals are diagnosed with PCLs, implementing proper follow-up plans is thought to impact the overall prognosis. Besides, the identification of these individuals is helpful in the detection of concurrent malignant lesions.^25^ As mentioned before, concomitant PC in IPMN includes synchronous and metachronous malignancy risk. In a study by Kamata et al., EUS outperformed conventional imaging in the early identification of PC concurrent with IPMN lesions.^76^ Higher risks of concomitant PC have been reported, for instance, in the presence of gastric-IPMN and GNAS mutation (see above).^77^

 PanIN lesions are the most commonly encountered lesions in the carcinogenesis of PC. High-grade PanIN is clinically significant and thus requires consideration for early detection of malignancy. Histologically, PanINs can demonstrate lobulocentric atrophic features similar to chronic pancreatitis.^40^ EUS has thus been shown to be impactful in the identification of such patterns, even in asymptomatic individuals. Additionally, about 95% of PanIN demonstrate KRAS mutations – a key molecular finding in PDAC.^78^ Thus, molecular findings have a potential role in the earlier detection of PanINs.^4^ Promises from EUS in identifying molecular findings, such as DNA abnormalities and KRAS mutation, have been mentioned in this regard.^75^ Das and others indicated the usefulness of monoclonal Antibodies in detecting high-grade PanINs. An accuracy of 90% was obtained for differentiating high-grade PanIN from low-risk lesions.^79^

 In an extensive review of the present challenges to the implementation of effective screening methods in PC by Chari and colleagues, the roles of circulating cellular markers have been re-emphasized.^29,80^ In addition to the mentioned markers of liquid biopsy, circulating tumor cells were shown to be influential in the detection and screening of different cancer types.^28^ Specifically, these markers enable early identification of PC. With further advancements in sampling and detection strategies, non-invasive methods are expected to change the existing limitations in the early detection of PC.^75^

###  Medical Management

 Currently, gemcitabine and nab-Paclitaxel combination therapy is the treatment of choice for advanced PC.^81^ Improving management guidelines for individuals with PCL is expected to make a direct impact on the global burden of PC. Among the PCLs, adjuvant therapy is currently indicated for cases with high-risk features of malignancy. Specifically, strong consensus exists over using systemic adjuvant chemotherapy for invasive IPMNs with positive lymph nodes.^23^ Right now, gemcitabine is the most commonly used agent for single adjuvant therapy in inv-IPMNs.

 Additionally, MCNs with malignant features are treated similarly to conventional.^8^ As mentioned by Wasif and colleagues, node positivity is a major adverse predictor for inv-IPMN prognosis. Specifically, node-positive invasive IPMNs demonstrate similar behaviors as node-positive PDAC –5-year survival rates ranging from 0% to 40%.^82^ Moreover, Alexander and colleagues indicated significantly poorer prognoses for node-positive individuals than for node-negative individuals (16 mo vs. 78 mo). Their results indicated significant improvement in survival measures following adjuvant chemotherapy only in the former group.^83^ McMillan and others revealed factors, including the TNM stage, greater than node positivity, margin positivity, and poor differentiation as predictors of response to adjuvant therapy.^84^ The presence of tubular histopathology has also been mentioned as a predictor for a good prognosis in the invasive IPMN lesion.^85^

 Despite many ongoing trials, the wide usage of targeted treatments in PC still needs to be amenable. Combinations of gemcitabine with molecular-targeted therapies were associated with limited improvements and severe toxicities.^10,86^ EGFR inhibitors are the most widely studied subgroup of targeted treatment used in PC – as combination targeted therapy. The addition of erlotinib – an EGFR inhibitor – to capecitabine was once demonstrated as the only combination therapy with improvements compared with single gemcitabine.^87^ Cetuximab is a competitive EGFR inhibitor acting on the surface receptor to prevent its function. Results obtained by a recent phase II randomized clinical trial performed by Liermann and colleagues indicated the addition of cetuximab as a safe option with improvements in local disease control. However, survival profiles achieved no significant difference compared with single chemoradiation therapy.^78,88^ Farnesyl transferase inhibitors were another group investigated in PC that targeted the KRAS pathway. The earliest studies indicated acceptable safety profiles for tipifarnib but no clinical benefit in survival measures compared with single chemo.^89^ Ongoing trials did not indicate promising results for molecular therapies in the clinical settings of PCLs.^90^ In summary, molecularly targeted therapies are still limited in the management of PC and its precursors.

 Vaccine-based therapy is one of the best examples of adjuvant targeted therapies, as they present specific tumor antigens to direct the immune system. In a cohort study, ras peptide-vaccinated individuals with GM-CSF adjuvant vaccines – GVAX – demonstrated survival benefits over those undergoing mere resections (20% compared with 0% in 10 years).^91^ A study by Abou-Alfa and co-workers showed acceptable tolerability with low immunogenic responses, limiting the utility of KRAS vaccines. Sequential studies demonstrated a failure of targeted KRAS monotherapy to effectively treat individuals with PC and put aside this adjuvant plan from the PC clinic.^92^ More recently, the telomerase peptide vaccine was investigated as a combination with adjuvant chemotherapy. Results of the phase III trial indicated no survival benefit compared with chemotherapy alone, limiting its clinical utility.^93^ Combination treatment strategies are thought to be most effective in overcoming immunohistological barriers in PC. According to a meta-analysis by Huang and colleagues, the combination of immunotherapy with chemotherapy induces several benefits in the clinical care of PC, compared with chemotherapy alone. Importantly, the analysis indicated a 1.17 risk ratio in the disease control rate and 0.87 in the hazard ratio related to progression-free survival.^94^ As there are many therapeutic options specific to the molecular mechanism of pancreatic malignancy,^95^ further investigation seems necessary.

## Conclusion

 Histomolecular studies have been shown to be promising tools for understanding the carcinogenesis pathway of PCLs. By providing the differentiating ability for benign and malignant lesions, they are helpful for proper patient selection and treatment specification. Additionally, addressing novel molecular-based methods is thought to help advance non-operative clinical decision-making. While still less is known about the targeted therapies in the setting of PC, by improving detection strategies and more efficient risk stratification, it is thought to improve the prognosis of individuals with pancreatic malignancy. As the models with less invasive PC entities, implementing such methods in individuals with PCLs is expected to unravel the risk-benefits of the treatments.
